# Clinical validation of a patient‐friendly saliva microsampling approach to monitor perampanel levels in people with epilepsy

**DOI:** 10.1002/epi.70302

**Published:** 2026-05-22

**Authors:** Valentina Franco, Francesca Dattrino, Michela Palmisani, Federica Tacchella, Elena Tartara, Valentina De Giorgis, Carlo Alberto Quaranta, Ludovica Pasca, Paola Rota, Emilio Russo, Andrea Romigi, Cecilie Johannessen Landmark, Guido Fedele, Cristina Tassorelli, Pierluigi Nicotera

**Affiliations:** ^1^ Department of Internal Medicine and Therapeutics University of Pavia Pavia Italy; ^2^ IRCCS Mondino Foundation Pavia Italy; ^3^ IRCCS Mondino Foundation, full member of European Reference Network EpiCARE Pavia Italy; ^4^ Department of Brain and Behavioral Sciences University of Pavia Pavia Italy; ^5^ Child Neurology and Psychiatry Unit, IRCCS Mondino Foundation, full member of European Reference Network EpiCARE Pavia Italy; ^6^ Department of Biomedical, Surgical, and Dental Sciences University of Milan Milan Italy; ^7^ Institute for Molecular and Translational Cardiology Milan Italy; ^8^ CRUISE Research Center, Science of Health Department Magna Græcia University Catanzaro Italy; ^9^ IRCCS Neuromed Mediterranean Neurological Institute Sleep Medicine Center Pozzilli Italy; ^10^ Departmental Faculty of Medicine Saint Camillus International University of Health Medical Sciences Rome Italy; ^11^ Program for Pharmacy, Faculty of Health Sciences Oslo Metropolitan University Oslo Norway; ^12^ Section for Clinical Pharmacology, Department of Pharmacology and National Center for Epilepsy full member of European Reference Network EpiCARE, Oslo University Hospital Oslo Norway; ^13^ Associazione Farmaceutici dell’Industria Milan Italy

**Keywords:** microsampling, perampanel, saliva, therapeutic drug monitoring

## Abstract

Perampanel (PER) is a third‐generation antiseizure medication (ASM) with a well‐established concentration–effect relationship. Saliva represents a non‐invasive alternative to plasma for therapeutic drug monitoring (TDM) of PER, as it reflects the free plasma fraction. Volumetric absorptive microsampling (VAMS) enables standardized, patient‐friendly saliva collection. This study aimed to clinically validate saliva‐VAMS by directly comparing it with conventional liquid saliva sampling for PER quantification. In a prospective study, saliva was collected from 11 people with epilepsy receiving PER therapy (dose range = 2–10 mg/day) using both VAMS and conventional saliva sampling. PER concentrations were measured by liquid chromatography coupled with tandem mass spectrometry and compared using repeated measures Bland–Altman analysis and Lin's concordance correlation coefficient to evaluate agreement between methods. PER concentrations measured in saliva‐VAMS showed excellent agreement with those obtained from conventional saliva sampling, with mean bias of −0.126 ng/mL (95% confidence interval = −0.582 to 0.330). No relevant constant or proportional bias was observed, and limits of agreement were within predefined acceptance criteria. Concordance between methods was high, supporting analytical equivalence. The demonstrated interchangeability between saliva‐VAMS and conventional saliva sampling supports adoption of saliva‐VAMS in clinical TDM workflows, enabling standardized fixed‐volume collection, simplified storage/transport, and potential home‐based sampling for remote dose optimization of PER and potentially other ASMs.

**Key points**

- Saliva‐VAMS provides a non‐invasive alternative for perampanel therapeutic drug monitoring.
- Perampanel concentrations obtained with saliva‐VAMS show strong agreement with conventional saliva sampling.
- No relevant constant or proportional bias was observed between the two saliva sampling methods.
- High concordance between methods supports the analytical equivalence of saliva‐VAMS and conventional sampling.
- Saliva‐VAMS enables standardized, patient‐friendly, and potentially home‐based sampling for perampanel monitoring.

## 1 INTRODUCTION

Perampanel (PER) is a third‐generation antiseizure medication (ASM) approved for the treatment of epilepsy. It acts as a selective, noncompetitive antagonist of α‐amino‐3‐hydroxy‐5‐methyl‐4‐isoxazole‐propionic acid (AMPA) receptors.^1^, ^2^ Evidence indicates a strong relationship between PER plasma concentrations, seizure control, and toxicity, supporting the importance of therapeutic drug monitoring (TDM).^3^, ^4^, ^5^ However, frequent venous blood sampling for TDM can be invasive and impractical, particularly in pediatric patients and in individuals requiring long‐term monitoring.^6^, ^7^ Saliva has emerged as a promising non‐invasive biological matrix for TDM, as salivary concentrations of PER correlate well with free, pharmacologically active plasma levels.^8^, ^9^ Volumetric absorptive microsampling (VAMS) offers a user‐friendly approach for collecting precise and reproducible volumes of saliva. The hydrophilic VAMS tip absorbs a fixed volume of sample, thereby improving analytical consistency, sample stability, and ease of transport, while also facilitating self‐collection.^10^, ^11^ This minimally invasive sampling strategy reduces patient discomfort, it is likely to increase adherence to monitoring protocols, and is particularly advantageous for vulnerable populations such as children and patients requiring repeated or long‐term TDM. Building on previous work by our group in the validation of the use of VAMS for saliva‐based PER monitoring,^12^ the present study aims to confirm the clinical applicability of this approach by directly comparing saliva‐VAMS with conventional liquid saliva collection. Demonstrating equivalence between these two sampling methods is essential to support the broader implementation of saliva‐VAMS in TDM, providing a fully non‐invasive, accurate, and patient‐friendly alternative to traditional plasma‐based monitoring for PER and potentially other ASMs. To this end, paired samples from the same individuals were analyzed using both collection methods, allowing a direct assessment of agreement. This approach provides evidence on the feasibility of adopting saliva‐VAMS for routine clinical TDM while maintaining accuracy, consistency, and clinical relevance of PER measurements. Establishing their interchangeability is essential to mitigate volume‐related variability and simplify sample handling, storage, and transport logistics, overall improving patient assistance.

## 2 MATERIALS AND METHODS

### 2.1 Chemicals and reagents

PER was provided by Eisai Co., Ltd. (Kashima, Japan). The internal standard, PER‐*d*_5_, and VAMS devices were obtained from B.S.N. (R&D Laboratory, Castelleone, Italy). Ultrapure water was produced using a Millipore‐Q‐plus system (Millipore, Milan, Italy). Liquid chromatography–mass spectrometry grade methanol, acetonitrile, and 99% formic acid were acquired from VWR International (Radnor, PA, USA), while dimethylsulfoxide (≥99.5%) was obtained from Sigma Aldrich (St. Louis, MO, USA). Drug‐free human saliva for method validation was collected from healthy volunteers.

### 2.2 Experimental

#### 2.2.1 Saliva collection and microsampling procedures

Saliva samples were collected from each subject in the morning under fasting conditions at steady state. Participants had been instructed to refrain from eating, drinking, or performing oral hygiene procedures for at least 30 min prior to collection. Two different sampling methods were employed. In the conventional approach, approximately 2 mL of unstimulated saliva was obtained using the passive drooling method. The sample was aspirated with a syringe, transferred into 2 mL polypropylene tubes, and subsequently stored at −20°C until analysis. VAMS sampling was performed on a small additional aliquot of the same saliva collected for the conventional method. After passive drooling into the collection tube, the VAMS tip was gently applied to the surface of the collected saliva, allowing absorption of 30 μL of fluid. The loaded VAMS devices were then dried at room temperature for 60 min before storage.

#### 2.2.2 Sample preparation

Calibrators and quality control samples were prepared by mixing 75 μL of saliva matrix with 75 μL of internal standard (PER‐*d*_*5*_, 140 ng/mL in methanol), 7.5 μL of a PER working solution, and 217.5 μL of methanol. Samples from people with epilepsy (PwE) were processed similarly, combining 75 μL of saliva with 75 μL of internal standard and 225 μL of methanol. Compared to the previously published manual protocol,^12^ increased volumes enabled direct liquid chromatography coupled with tandem mass spectrometry (LC–MS/MS) injection without evaporation, streamlining the workflow and preserving analyte stability. Sample preparation for conventional saliva, including mixing, centrifugation, filtration, and transfer, was fully automated on a high‐throughput Biomek SAMI EX workstation (Beckman Coulter Life Sciences, Indianapolis, IN, USA). The system integrates a Biomek i7 liquid‐handling system, a Positive Pressure Unit (PPU) V4 system, a microplate centrifuge, a SCARA robotic arm for plate transfer, and a Cytomat 10 ambient storage unit. Supernatants obtained after centrifugation were filtered using Acroprep Advance 30 K Omega plates (Cytiva, Milan, Italy) on the PPU V4 system (1500 mbar filter pressure, 3000 mbar clamp pressure, 10 min; Amplius GmbH, Rostock, Germany). Filtrates were directly transferred to the LC–MS/MS autosampler, and 20 μL was injected for analysis. VAMS samples were subjected to the procedure described by Palmisani et al.^12^

#### 2.2.3 LC–MS/MS analysis

PER quantification was performed using a validated LC–MS/MS method, as described by Palmisani et al.^12^ The analysis employed a SCIEX ExionLC 100 liquid chromatography system coupled with a SCIEX API 3200 QTRAP triple quadrupole mass spectrometer (Applied Biosystems SCIEX, Darmstadt, Germany) equipped with an electrospray ionization source operating in positive ion mode. Chromatographic separation was carried out using a C18 column (Onyx, 100 × 3 mm inner diameter, Phenomenex, Torrance, CA, USA) maintained at 25°C. The mobile phases consisted of water with 0.1% formic acid (phase A) and methanol (phase B), with a flow rate of 0.9 mL/min and a programmed gradient elution. Mass spectrometry data were acquired in multiple reaction monitoring mode.

#### 2.2.4 Clinical applicability and method comparison

Saliva and VAMS samples were collected from a total of 11 PwE (20 pairs) treated with a stable dose of PER in combination with other ASMs. The study population included the following: one subject receiving a dose of 4 mg/day; a second subject treated with three different doses (6, 8, and 4 mg/day); a third and a fourth subject receiving a stable dose of 10 mg/day and 2 mg/day, respectively; and a fifth subject treated with two different dosages (4 and 6 mg/day). Furthermore, six additional VAMS/saliva sample pairs were collected from the remaining subjects (Table 1).

**TABLE 1:** Clinical and demographic characteristics of the PwE included in the analysis.

PwE ID	Age, years	PER dosage, mg/day	Concomitant ASMs
1	50	4	CBZ, PB, TPM
1	50	4	CBZ, PB, TPM
1	50	4	CBZ, PB, TPM
2	18	6	CBD, LTG, PB, TPM
2	19	8	CBD, LTG, PB, TPM
2	20	4	LTG, PB
2	21	4	LTG, PB, STC
3	38	4	CBZ, PB, VGB
4	19	6	ESM
5	21	10	VPA
5	21	10	VPA
6	26	2	LCM, LEV
6	27	2	LCM, LEV
7	15	4	FBM, VPA
7	15	6	FBM, VPA
7	15	6	FBM, VPA
8	64	8	LCM, PB, TPM
9	55	10	LCM, LEV
10	54	4	LCM
11	29	8	LEV, LTG, PRM

Abbreviations: ASM, antiseizure medication; CBD, cannabidiol; CBZ, carbamazepine; ESM, ethosuximide; FBM, felbamate; LCM, lacosamide; LEV, levetiracetam; LTG, lamotrigine; PB, phenobarbital; PER, perampanel; PRM, primidone; PwE, people with epilepsy; STC, soticlestat; TPM, topiramate; VGB, vigabatrin; VPA, valproic acid.

Some PwE provided multiple samples at the same PER dose. Participants were recruited from the Child Neurology and Psychiatry Unit and the Epilepsy Center of the IRCCS Mondino Foundation in Pavia, Italy. The study was conducted in accordance with the Declaration of Helsinki and was approved by the Ethics Committee of the IRCCS Policlinico San Matteo, Pavia (reference no.: P‐20170012031). Written informed consent was obtained from all participants or their legal guardians prior to inclusion. Comparisons between PER concentrations in saliva and VAMS were performed using Lin's concordance correlation coefficient to evaluate agreement. Repeated measures Bland–Altman analysis was employed to assess concordance between the two measurement techniques by analyzing the differences in concentrations obtained from VAMS and saliva relative to their mean values. Limits of agreement (LoA) were estimated as the mean difference ± 1.96 times the standard deviation, and corresponding 95% confidence intervals (CIs) were calculated for both the mean bias and the LoA. PER concentrations were expressed in ng/mL. All statistical analyses were conducted using R software (version 4.2.3, R Foundation for Statistical Computing, Vienna, Austria) with the R Studio graphical interface (version 2024.4.2.764, Posit team, Posit Software, PBC, Boston, MA, USA).

## 3 RESULTS

### 3.1 Comparative evaluation of saliva‐VAMS and conventional saliva sampling

The analysis was conducted on a total of 20 paired saliva samples, analyzed using two different sampling methods: saliva‐VAMS and conventional saliva. Results obtained using VAMS were compared with results obtained from direct saliva measurements.

**FIGURE 1:**
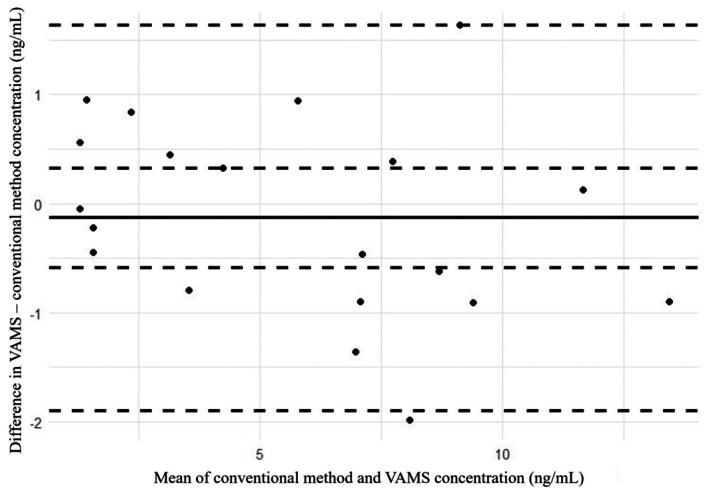
Repeated measures Bland–Altman analysis of perampanel concentrations comparing volumetric absorptive microsampling (VAMS) saliva and conventional saliva (*n* = 20 pairs). The x‐axis represents the mean concentration of each pair, and the y‐axis shows the difference between methods. The solid line indicates the mean bias, and the outer dashed lines represent the limits of agreement. The inner dashed lines indicate the 95% confidence interval of the mean bias. Estimates were obtained accounting for repeated measurements.

Figure 1 presents the repeated measures Bland–Altman analysis performed on 20 paired VAMS and saliva samples. The analysis showed strong agreement between methods, with a mean bias of −0.126 ng/mL (95% CI = −0.582–0.330). The lower LoA was −1.892 ng/mL (95% CI = −2.625–1.159), and the upper LoA was 1.640 ng/mL (95% CI = 0.907–2.373). Lin's concordance correlation coefficient, calculated using subject‐level mean values, was 0.980 (95% CI = 0.944–0.993), indicating excellent agreement between VAMS and the conventional method.

## 4 DISCUSSION

This study provides a clinical validation of saliva‐based VAMS for PER TDM by directly comparing it with conventional liquid saliva collection. The results demonstrate an excellent agreement between the two sampling approaches, supporting their analytical and clinical interchangeability. Statistical analyses consistently indicated the absence of relevant bias between VAMS and conventional saliva method. Repeated measures Bland–Altman analysis confirmed strong agreement, showing a minimal mean bias and narrow LoA within predefined acceptance criteria. Lin's concordance correlation coefficient approached unity, indicating almost perfect concordance between methods. Collectively, these findings demonstrate that saliva‐VAMS provides PER concentration values using very small sample volumes, fully equivalent to those obtained from conventional saliva sampling. The clinical relevance of these findings is supported by the well‐established concentration–effect relationship of PER. Previous studies have shown that PER plasma concentrations are strongly associated with seizure control and tolerability, highlighting the potential role of TDM in optimizing treatment.^3^, ^4^, ^5^, ^13^, ^14^ Saliva has emerged as a reliable alternative matrix for TDM of several ASMs, including PER, as salivary concentrations reflect the free, pharmacologically active fraction in plasma.^8^, ^9^ Despite these advantages, conventional liquid saliva collection may present practical limitations, such as variability in collected volume, difficulties in handling viscous samples, and the need for appropriate storage and transport conditions. VAMS addresses many of these challenges by enabling the collection of a fixed, small volume of saliva, thereby improving analytical consistency, sample stability, and ease of handling.^10^, ^11^ In addition, the drying of VAMS samples at room temperature enhances analyte stability and reduces dependence on cold‐chain logistics, which is particularly advantageous in decentralized or resource‐limited settings. The present findings extend and confirm previous work by our group on the analytical feasibility of saliva‐VAMS for PER monitoring^12^ by providing direct clinical evidence of equivalence with conventional saliva sampling. This head‐to‐head comparison is a critical step toward clinical implementation, as demonstration of interchangeability is increasingly required when alternative sampling strategies are proposed for TDM.^15^ The paired‐sample design adopted in this study minimized interindividual variability and allowed a robust assessment of agreement across different PER doses and concentration ranges. In this context, although conventional saliva samples were processed using an automated platform and VAMS samples manually, the agreement analyses did not suggest the presence of systematic or proportional bias. From a patient‐centered perspective, saliva‐VAMS offers clear advantages, particularly for populations in whom repeated venipuncture is undesirable or impractical, such as pediatric patients and individuals requiring long‐term monitoring. The ability to obtain accurate measurements from very small volumes of saliva makes this approach highly suitable for self‐collection, remote monitoring, and patient‐centered care models, while also ensuring feasibility in clinically compromised patients due to its practicality and ease of handling. These features may improve adherence to monitoring and support more individualized dose adjustments, in line with current best‐practice recommendations for ASM management.^6^, ^7^ Some limitations should be acknowledged. The number of PwE included was relatively limited, which may somewhat constrain the generalizability of the findings to different patient populations, although it was sufficient for method comparison and agreement analyses. Furthermore, all samples were collected under supervised, steady‐state conditions. Future studies should evaluate the performance of saliva‐VAMS in unsupervised home‐sampling settings and during longitudinal follow‐up, as well as confirm these findings in larger and more diverse cohorts and explore its potential extension to other ASMs and clinical settings. Nonetheless, the excellent agreement observed in this study strongly supports the robustness and clinical applicability of saliva‐VAMS for PER TDM.

## 5 CONCLUSIONS

PER concentrations measured in saliva using VAMS were fully comparable to those obtained from conventional liquid saliva collection. The high level of agreement confirms the analytical validity and interchangeability of VAMS for PER TDM. This non‐invasive, patient‐friendly approach combines ease of use with the ability to obtain accurate measurements from very small sample volumes, making it well‐suited for routine clinical practice as well as remote or home‐based monitoring. The successful validation of saliva‐VAMS for PER also suggests potential applicability to other ASMs, supporting broader implementation of microsampling strategies in epilepsy care.

## AUTHOR CONTRIBUTIONS

*Conceptualization:* Valentina Franco, Pierluigi Nicotera, Cristina Tassorelli. *Methodology:* Francesca Dattrino, Valentina Franco, Cecilie Johannessen Landmark, Michela Palmisani, Paola Rota, Federica Tacchella. *Formal analysis:* Francesca Dattrino, Guido Fedele, Valentina Franco, Michela Palmisani, Federica Tacchella. *Investigation:* Valentina De Giorgis, Ludovica Pasca, Carlo Alberto Quaranta, Andrea Romigi, Emilio Russo, Elena Tartara, Cristina Tassorelli. *Data curation:* Francesca Dattrino, Guido Fedele, Valentina Franco, Michela Palmisani, Paola Rota, Federica Tacchella. *Writing—original draft preparation:* Francesca Dattrino, Valentina Franco, Cecilie Johannessen Landmark, Pierluigi Nicotera, Michela Palmisani, Emilio Russo, Federica Tacchella. *Writing—review and editing:* all authors. *Visualization:* all authors. *Supervision:* Valentina Franco, Pierluigi Nicotera, Cristina Tassorelli. *Project administration:* Valentina Franco. *Funding acquisition:* Valentina Franco, Pierluigi Nicotera.

## FUNDING INFORMATION

This research was funded by a grant from the Italian Ministry of Health (Ricerca Corrente 2025‐26) and by the Italian Ministry of Health through the “5 × 1000” program (IRCCS Mondino Foundation, Pavia, Italy).

## CONFLICT OF INTEREST STATEMENT

V.D.G. has served on scientific advisory boards for Longboard Pharmaceuticals and has received research funding, speaker fees, and consultancy fees from Jazz Pharmaceuticals and speaker or consultancy fees from Novartis, Nutricia, Vitaflo, and Dr. Schär Kanso, all unrelated to the present study. C.J.L. has received speakers' or expert group honoraria from Angelini, Eisai, Jazz Pharmaceuticals, and UCB Pharma. E.R. has received speaker honoraria, research support, and advisory board fees from Angelini Pharma, Arvelle Therapeutics, Eisai, Ethypharm, GW Pharmaceuticals, Jazz Pharmaceuticals, Kolfarma, Lundbeck, Pfizer, and UCB. E.T. has received speaker or consultancy fees from Angelini Pharma and Ecupharma. Over the past 3 years, C.T. has received funding and/or investigational product support from AbbVie and Novartis for an investigator‐initiated study. She has received advisory board fees from AbbVie, Dompé, Eli Lilly, Ipsen, Lundbeck, Medscape, Pfizer, and Teva; speaker honoraria from AbbVie, Eli Lilly, Lundbeck, Pfizer, and Teva; travel support from AbbVie, Eli Lilly, Dompé, Ipsen, Teva, Lundbeck, and Pfizer. She has also served as principal investigator in clinical trials sponsored by AbbVie, Biohaven, Eli Lilly, Ipsen, Lundbeck, Pfizer, and Teva and has received research grants from the European Commission, the Italian Ministry of Health, and the Italian Ministry of University and Research. The remaining authors declare no conflicts of interest. We confirm that we have read the Journal's position on issues involved in ethical publication and affirm that this report is consistent with those guidelines.

## Data Availability

The data supporting the findings of this study are available from the corresponding author upon reasonable request.
